# Analysis of Stress Indicators for Evaluation of Animal Welfare and Meat Quality in Traditional and Jewish Slaughtering

**DOI:** 10.3390/ani8040043

**Published:** 2018-03-21

**Authors:** Giancarlo Bozzo, Roberta Barrasso, Patrizia Marchetti, Rocco Roma, Giorgio Samoilis, Giuseppina Tantillo, Edmondo Ceci

**Affiliations:** 1 Department of Veterinary Medicine, University of Bari Aldo Moro, Strada Provinciale per Casamassima km 3, 70010 Valenzano (BA), Italy; roberta.barrasso@uniba.it (R.B.); patrizia.marchetti@uniba.it (P.M.); giuseppina.tantillo@uniba.it (G.T.); edmondo.ceci@uniba.it (E.C.); 2 Department of Agricultural and Environmental Science, University of Bari Aldo Moro, 70125 Bari (BA), Italy; rocco.roma@uniba.it; 3 Slaughterhouse, Freelance Veterinary, Str. Prov. 70027 Palo del Colle (BA), Italy; g.samoilis@sicilianispa.com

**Keywords:** cortisol, dopamine, norepinephrine, epinephrine, animal welfare

## Abstract

**Simple Summary:**

Cortisol and catecholamines (dopamine, norepinephrine and epinephrine) are released in response to stress and directly stimulate glycogen mobilization, thus influencing meat acidification. The aim of the study was to estimate and compare these stress indicators to evaluate the welfare of beef cattle, subjected to either traditional slaughtering (with stunning) or to slaughtering with religious Jewish rite (without stunning). Significant differences in plasma cortisol and catecholamine levels were observed during exsanguination by monitoring animals in the pre-slaughtering (before and after transportation) and slaughtering phases. Cortisol, dopamine and norepinephrine, but not epinephrine, were markedly higher in the animals slaughtered by the religious rite. Pursuing animal welfare in the religious slaughtering procedures could produce advantages in terms of hygiene, organoleptic quality and shelf life of meat.

**Abstract:**

Sixty Charolais male beef cattle of eight months of age were divided into two groups according to the slaughtering method, i.e., traditional or Kosher (religious Jewish rite). The aim of the study was to detect and compare the plasma concentrations of cortisol and catecholamines (dopamine, norepinephrine and epinephrine), by Elisa and HPLC test. These four stress indicators were evaluated during three different stages of each animal productive life: on the farm (step 1), after transportation (step 2) and during bleeding (step 3). The patterns of the parameters measured were similar and, interestingly, revealed significant changes throughout the three steps considered. The greatest variation between the two methods of slaughtering was observed in step 3, where we found a statistically significant difference with all the parameters except epinephrine. In the animals slaughtered by the religious rite, cortisol, dopamine, norepinephrine and epinephrine were 68.70 ± 30.61 nmol/L; 868.43 ± 508.52 ng/L; 3776.20 ± 1918.44 ng/L; and 4352.20 ± 3730.15 ng/L, respectively, versus 45.08 ± 14.15 nmol/L; 513.87 ± 286.32 ng/L; 3425.57 ± 1777.39 ng/L; and 3279.97 ± 1954.53 ng/L, respectively, in the other animals. This suggests that the animals slaughtered by the Kosher rite are subjected to higher stress conditions at the exsanguination phase. The animals slaughtered by the religious Jewish rite showed lower cortisol and catecholamine levels on the farm (step 1) and after transportation to the slaughterhouse (step 2). This was likely because the animals selected at the end of step 1 by the Rabbis for the religious rite are usually the most docile and gentle.

## 1. Introduction

The hypothalamic–pituitary–adrenocortical (HPA) axis releases glucocorticoids, such as cortisol, as part of the endocrine mechanism for self-protection of the body in presence of a stressor. The quantification of cortisol or its metabolites is a physiological indicator for the stress assessment [1]. Several studies have focused on the evaluation of cortisol or cortisol metabolites levels in plasma, faeces, urine, saliva and milk [2]. It is well known that both the sympathetic and the hypothalamic–pituitary–adrenal axis are involved when animals are exposed to stressful situations [3]. The activation of the sympathetic axis responds to short-term stress through the release of catecholamines (epinephrine and norepinephrine) into the blood stream [4,5]. Cortisol plays a role in acute or chronic stress and it is able to mobilize the energy reserves through the conversion of glycogen into energy [6]. Stress increases cortisol concentration in blood and triggers depletion of glycogen reserves in muscles [7]. This can lead to a decrease in post-mortem lactic acid production and cause a high pH of the meat [8]. Cortisol is used as a physiological indicator in dairy cows, pigs and goats [9]. Cortisol and catecholamines are released in response to stress and directly stimulate glycogen mobilization [10]. Physiological stress responses (animal welfare indicators) were also correlated to plasma catecholamines (epinephrine and norepinephrine) and to biochemical and hematological parameters [11]. Catecholamines (dopamine, norepinephrine and epinephrine) are important neurotransmitters of the sympathetic nervous system [12]. Under normal physiological conditions, catecholamines are released from the adrenal medulla to maintain body homeostasis and to regulate several body functions including maintenance of blood pressure [13]. However, under stressful situations, high concentrations of catecholamines are discharged into the bloodstream in preparation for the possibility of rapid energy expenditure [13].

During stressful situations, the secretion of catecholamine and glucocorticoids stimulates hepatic glycogenolysis leading to an increase of glucose levels [13,14,15] and therefore to a reduction of post-mortem lactic acid production [16]. Hepatic glycogenolysis result in glycogen depletion before slaughtering, elevated ultimate pH (pHu) and unacceptable conversion from muscle to meat [11,17]. Furthermore, exercise and psychological stress shortly before slaughtering increase muscle metabolic activity, which may continue after death, resulting in faster post-mortem pH decline and thus decreased meat quality [18]. When an animal bleeds out, there is a fall in pressure, which activates the sympathetic adrenal medullary nervous system, resulting in the release of epinephrine and norepinephrine from the sympathetic terminations [19].

Kosher slaughter, known as *shechitàh*, is the only method used by the Jewish Community. Precise precepts define which animals are kosher and only those selected in the breeding are suitable for religious slaughtering [20]. The religious slaughter is performed according to precise ritual rules (blessings or invocations) [21]. Religious rules may inflict unjustified suffering to the animals, which are immobilized and killed without stunning [22]. The rite requires the killing of the animal by cutting the trachea, the esophagus and the blood vessels with a very sharp blade. This procedure is done to cause a rapid drop of brain blood pressure and the loss of consciousness, to render the animal insensitive to pain. Moreover, the procedure is intended to exsanguinate promptly the animal. The cut must be incised with a back and forth motion without violating one of the five major prohibited techniques (pause, pressure, stabbing, slanting or tearing) or various other detailed rules [23]. Post-procedure requirements involve that the animal is checked for the presence of eventual lesions, especially in the lungs and liver. According to the number and type of lesions found in the lungs and liver, the carcasses will then be classified as *chalak* (or *glatt*), *kosher* and *terif* [24,25]. After inspection of the organs, some portions of fat and organs such as the kidneys, the intestines and the sciatic nerve, are removed through a process called *nikkur*. Since blood is not considered edible, all large arteries and veins are removed, as well as any bruised meat or coagulated blood; the meat is then purged of all remaining blood through the koshering process [20,21].

We have previously evaluated the plasma cortisol levels and found significant variations during the various phases of slaughtering between traditional and *Kosher* slaughtering [26]. The aim of the study was to estimate and compare other parameters (catecholamines) considered as stress indicators, in relation to the variations of cortisol, in the various phases of beef cattle slaughtering, following either traditional procedures, which include stunning, or the religious Jewish rite, where stunning is not contemplated.

## 2. Materials and Methods

### 2.1. Ethical Statement

The experimental procedures were approved by the ethical committee of the Department of Veterinary Medicine at Bari (Italy) University (Protocol n.: 605-III/13, 4 April 2017).

### 2.2. Sampling

The study, resulting from a partnership between the Food Safety Section of the Department of Veterinary Medicine at Bari University and a slaughterhouse in Apulia region (Southern Italy), was conducted in the period between April and June 2017.

The study was carried out on a total of sixty Charolais male beef cattle of eight months of age, bred in a free paddock outdoors. After a transport time of about 45 min, all the animals arrived at the same slaughtering establishment. Before slaughtering, all the animals were kept in the lairage facilities for a period of about 30 min.

The animals were divided into two experimental groups, each one consisting of thirty individuals, to verify the whole chain production, and to ensure a product conforming to Jewish rules. Thirty animals (group A) were selected by the Rabbis responsible for the Shechitàh Committee directly on the farm. The selection criteria were decided by the Rabbis on the basis of his experience and tradition. The animals selected for the religious rite were usually the most docile and gentle, although these selection criteria were disclosed only at the end of the experiment. This group of animals were slaughtered the day after the animals slaughtered with the conventional procedure, but always at the same hour. At the slaughterhouse the Rabbis restrained the animals in a full inversion rotary pen. After this step, the animals were slaughtered by authorized slaughter-men of the Jewish faith by a perfectly clean incision, using a *Chalaf* (Shechitàh knife) through the structures at the front of the neck: trachea, oesophagus, carotid arteries and jugular veins.

Conversely, the other thirty animals (group B) were slaughtered after stunning by captive bolt gun, which causes immediate loss of consciousness, making the animals insensible to pain until death supervenes through exsanguination, as required from the Council regulation (EC) N° 1099/2009 on the protection of animals at the time of killing [27]. These thirty calves were from the same farm as the animals slaughtered by the *Kosher* rite and they were randomly selected by the operators.

The plasma levels of cortisol and catecholamines (dopamine, norepinephrine and epinephrine) were evaluated during three different stages of animal productive life: on the farm during growth, one week before slaughtering (step 1); after transportation, in the lairage facilities of the slaughterhouse, thirty minutes after the animal discharge (step 2); and finally, during bleeding (step 3).

Blood samples of the first two steps were collected from the jugular vein, both at 6:00 a.m. to exclude a circadian variation. In step 3 the blood samples were collected during the exsanguination phase, which was carried out 15–30 min after step-2 sampling. The blood samples were collected in vacutainer test tubes containing ethylenediaminotetracetic-acid (EDTA) and stored in ice at 0 °C for no longer than 60 min, avoiding freezing, before submitting to the reference laboratory.

### 2.3. Plasma Cortisol—Elisa Test

Plasma cortisol was determined as described in a previous study [26]. Briefly, the cortisol ELISA immunoassay test (Bovine-Cortisol ELISA; My-Bio-Source, San Diego, CA, USA) was used following the manufacturer’s guidelines.

All reagents were kept at room temperature (25–28 °C) for 30–40 min before being reconstituted. Enzyme conjugate was stored at −20 °C until use. Highly concentrated samples were diluted with sample diluent (e.g., 1:5 or 1:10) to obtain a readable range on the curve.

In the first step, 50 μL of standard was added to each standard well, 50 μL of plasma to each sample well and 50 μL of sample diluent to each blank/control well. Standards, samples and diluent were added in duplicate to the plate.

In the second step, 100 μL of HRP% (Horseradish Peroxidase) conjugate reagent was added to each well and incubated for 60 min at 37 °C. In the third step, the plate was washed 4 times with a wash solution (250–300 μL per well) and then residual liquids were carefully removed. In the fourth step, 100 μL of the colour reagent tetramethylbenzidine (TMB) were added to each well and the plate was incubated for 30 min at 18–25 °C without shaking. The reaction was stopped by adding 100 μL of 1M H_2_SO_4_ to each well and mixing gently for 1–2 min. Shortly after stopping the reaction, the optical density (OD) of each was determined using a microplate reader with a wavelength of 450 nm, 540 nm or 570 nm.

The mean of the readings of duplicates for each standard and sample was calculated, and the average OD of the blank was subtracted. A standard curve was created using computer software capable of generating a four-parameter logistic (4-PL) curve-fit.

The minimum detectable dose of bovine cortisol (sensitivity) was ≥0.049 ng/mL. The detection range was 0.049–200 ng/mL. No significant cross reactivity or interference between bovine cortisol and analogues was observed. Intra-assay precision CV (%) was <8%, while inter-assay precision CV (%) was <10%.

### 2.4. Plasma Catecholamine—HPLC Test

#### 2.4.1. Description

The catecholamines plasma kit (ClinRep; Recipe Chemicals and Instruments GmbH, Munchen, Germany) was designed for the quantitative determination of catecholamines from plasma with High Performance Liquid Chromatography (HPLC). HPLC with electrochemical detection was established as a reliable and sensitive method for the determination of catecholamines in plasma.

#### 2.4.2. Reconstitution of the Calibrator and Controls

The ClinCal Plasma Calibrator and the ClinChel Plasma Controls were lyophilized and needed to be reconstituted prior to use with deionised water.

#### 2.4.3. Assay Procedure

One milliliter of plasma was pipetted into the sample preparation column (directly onto the aluminium oxide suspension) and subsequently 50 µL of internal standard was added. The column was closed and it was shaken upside down for 10 min. The column was slightly tapped on to transfer these residues back to the bottom of the frit column. Then, the upper and lower caps of the sample preparation column were removed, the supernatant was aspirated (with a vacuum station) and the effluent was discarded. One milliliter of washing solution was put in the sample preparation column and then the washing solution was aspirated. The washing procedure was carried out three times and, after these steps, the effluent was discarded. The elution vial was plugged onto the sample preparation column and 120 µL of eluting reagent was pipetted into the column. Afterwards it was mixed for 1 min on a vortex mixer. Subsequently the eluate was centrifuged through the column into the elution vial (1 min a 1000 rpm). The elution vial may be used for subsequent sample injection in the auto-sampler.

#### 2.4.4. HPLC System

The HPLC pump flow rate was 1 mL/min. The analytical column was installed in the column heater at 25 °C. Auto-sampler injection volume: 40 µL (prepared sample, calibrator or controls). Injection interval: 15 min. Electrochemical Detector parameters: Potential 500 mV; Sensitivity 10 nA; Filter setting 0.2 Hz.

#### 2.4.5. Calculation of Results

The concentration of the analytes was calculated with the internal standard method via the peak areas. According to the internal standard method, each sample was spiked with a so-called “internal standard” prior to the sample preparation. The internal standard was similar to the analytes in terms of behavior during sample preparation and chromatography. Hence, any losses during the sample preparation could be determined by calculating the recovery. The extrapolation to 100% recovery allowed the determination of the concentration of the unknown substances in the sample.

#### 2.4.6. Performances

(i) Linearity: Dopamine 30–2500 ng/L (lower limit of detection: 15 ng/L; lower limit of quantitation: 30 ng/L). (ii) Linearity: Norepinephrine 15–2500 ng/L (lower limit of detection: 8 ng/L; lower limit of quantitation: 15 ng/L). (iii) Linearity: Epinephrine 15–2500 ng/L (lower limit of detection: 8 ng/L; lower limit of quantitation: 15 ng/L).

### 2.5. Statistical Analysis

To describe the changes in the concentration of plasma cortisol and catecholamines during the three stages of animal productive life, a statistical descriptive analysis based on central tendency and concentration indexes was carried out for each group of animals and for the four parameters considered. A first evaluation of the difference and of the significance of the differences observed within the two groups and in the three phases was verified by a Generalized Linear Model (GLM) with repeated measures. In particular, the Mauchly’s test of sphericity was used to evaluate if there were any significant differences in the variance of the mean values in the three steps studied. This analysis was carried out since the three samples of each animal were not independent, but they were represented by the same calves in the three steps considered. Moreover, three different increasing rates were evaluated starting from the observation of different tendency in cortisol and catecholamine concentrations between the two groups of animals: (i) between cattle farm and lairage facilities; (ii) between lairage facilities and the exsanguination phase; (iii) between cattle farm and the exsanguination phase, i.e., for the whole chain production.

The mean values of the two groups and of the four parameters evaluated in this study were compared by one-way analysis of variance.

## 3. Results

The mean values, the standard deviation and the significance of the differences of the physiological indicators are shown in the Table 1. The cortisol levels were determined previously [26]. Cortisol was lower in group A than in group B in step 1 (F: 0.648; *p* = 0.424) and 2 (F: 14.263; *p* = 0.000); on the other hand, cortisol levels were higher in the animals of group A than in the animals of group B (F: 16.021; *p* = 0.000) in step 3. The same trend was also found for the other three parameters object of our study. In fact, dopamine was lower in group A than in group B in step 1 (F: 1.65; *p* = 0.20) and 2 (F: 3.53; *p* = 0.07); conversely, dopamine levels were higher in the animals of group A than in the animals of group B (F: 5.82; *p* = 0.02) after the exsanguination phase. Plasma norepinephrine was lower in the animals slaughtered by Jewish religious rite compared to the animals slaughtered by traditional method in step 1 (F: 0.08; *p* = 0.78) and 2 (F: 2.98; *p* = 0.09); on the other hand, norepinephrine levels were higher in the animals of group A than in the animals of group B (F: 8.19; *p* = 0.01) in step 3. Finally, plasma epinephrine was lower in group A compared to group B in step 1 (F: 0.01; *p* = 0.93) and 2 (F: 1.03; *p* = 0.31); conversely, epinephrine was higher in the animals of group A than in the animals of group B (F: 1.96; *p* = 0.17) after the exsanguination phase. Therefore, epinephrine values showed the greatest variation between the farm (step 1) and the exsanguination phase (step 3), increasing 69.69 times in the animals slaughtered by Jewish religious rite (Table 2).

Mauchly’s test of sphericity was significant (*p* < 0.005; Sig = 0.000) and this implied that the variance of the mean values in the three steps tended to remain constant for all the parameters studied. This showed that the repetitiveness of the measures did not influence the observed mean values. Having assumed the sphericity of the model, the differences were significant for each parameter in the three steps: cortisol (F: 203.482; *p* = 0.000), dopamine (F: 99.154; *p* = 0.000), norepinephrine (F: 179.129; *p* = 0.000) and epinephrine (F: 84.261; *p* = 0.000).

As regards the effect of the two different methods of slaughtering, cortisol and dopamine were particularly significant (F: 17.551; *p* = 0.000 and F: 11.252; *p* = 0.000, respectively), while norepinephrine (F: 0.811; *p* = 0.447) and epinephrine (F: 2.244; *p* = 0.111) were less significant.

## 4. Discussion

In this study we monitored animal stress during the slaughtering phases, trying to compare traditional and religious procedures. The patterns of the stress indicators (plasma cortisol, dopamine, norepinephrine and epinephrine) measured for monitoring the stress of the animals were similar and, interestingly, revealed significant changes among the three steps considered, i.e., on the farm (step 1), after transportation (step 2) and finally during bleeding (step 3).

The greater variation between the two methods of slaughtering was observed in step 3, where we found a statistically significant difference with all the parameters but epinephrine. In particular, of the four parameters examined, the plasma cortisol levels showed the greatest variation and significance by statistical analysis (Table 1). For instance, animals slaughtered according to the rules imposed by the religious Jewish rite, appeared to have lower cortisol and catecholamine levels when they were on the farm (step 1) and after transportation to the slaughterhouse (step 2) than animals subjected to traditional slaughtering. Differences in step 1 were unexpected, as the animals used for this study were all from the same farm and the different procedures of step 2 and 3 are unlikely to influence or pre-determine the status of the animals in step 1. When interrogating the operators in the farm, they provided us with a possible explanation. The animals selected at the end of step 1 by the Rabbis for the religious rite are usually the most docile and gentle. Therefore, their docile temperament could account for the differences observed in the levels of cortisol and catecholamines. On the other hand, the levels of the stress indicators determined immediately after the exsanguination phase (step 3) appeared much higher in the animals slaughtered by the religious rite than in traditionally slaughtered animals.

In particular, plasma cortisol levels showed a similar trend in all three steps of our experimental study and this mirrors the results described in traditionally slaughtered animals after stunning [8]. Conversely, the average plasma cortisol values observed in this study were lower than those reported in the literature [28,29]. The plasma dopamine values of the calves slaughtered by traditional method, detected in steps 1 and 2, were slightly higher than the values observed in the animals slaughtered by the Jewish religious rite. The dopamine levels observed during the exsanguination phase (step 3) were much higher in the animals slaughtered without stunning than in those ones slaughtered by traditional method.

The animals of group A were restrained using a rotary pen by which the animals were turned upside down for the cutting procedure. This phase is likely very stressful for the animals. As indicated by the statistical analysis between the two groups of animals, the two slaughtering methods appeared to change markedly at the exsanguination phase in the levels of plasma cortisol and, to a lesser extent, of norepinephrine and dopamine and of epinephrine (Table 1). Regarding epinephrine, the variation between the values found in the Jewish religious rite and the traditional slaughtering was few significant (Table 1), indicating that epinephrine is the catecholamine released more frequently and massively in the events which involve the fight or flight reaction of the Autonomic Nervous System (ANS) [30], regardless of the slaughtering method. Conversely, cortisol showed the greatest variation by comparing the two slaughtering methods, with a difference of about of about 16 times between the two bovine groups.

As observed in previous studies [31], our investigation confirms that the slaughtering method influences the plasma cortisol levels, which play a central role in the meat acidification. Moreover, during stressful situations, the secretion of catecholamine and glucocorticoids stimulates hepatic glycogenolysis, leading to an increase of glucose levels [14,15]. After exsanguination, the muscles develop anoxia conditions and anaerobic glycolysis is triggered, during which glycogen is hydrolyzed into lactic acid. Therefore, meat pH decreases from 7 to 5.5, an essential condition for the reduction of bacterial growth [32]. Among stress-induced changes, epinephrine is most likely to play an important role in the determination of meat quality by increasing the pH value, which may affect the correct conversion of muscle into meat [5,33,34].

Moreover, beef meat with pHu values higher than 6.0 is not desirable because of its decreased shelf life, dark colour, high variation in tenderness, increased Water Holding Capacity (WHC) and poor palatability [8,35,36]. According to the kosher process, the post-slaughtering treatment of the carcasses in saline solution is critical to remove blood from the muscle tissues, although according to Farouk et al. (2014), this practice is able to affect negatively the colour, the flavor and the organoleptic quality of meat and it may change the oxidative processes [20].

Cortisol plays a central role in the process of protein and fat degradation. Moreover, the increased levels of cortisol, even in the late slaughtering phases, may alter the organoleptic characteristics of the meat, such as a considerable decrease in marbling fat, which affects negatively meat flavor and tenderness [19].

## 5. Conclusions

The animals selected for *Kosher* showed lower levels of cortisol and catecholamine before and after transportation to the slaughterhouse, likely because of their more docile temperament was the rationale for their selection by the Rabbis. The levels of the stress indicators considerably increased at the exsanguination phase in the same animals. A possible reason for this was the fact that kosher animals are not stunned and they are restrained using a rotary pen, by which they are turned upside down for the cutting procedure, thus stressing considerably the animals.

Our experimental design, whilst providing useful insights into the stress factors/conditions of religious procedure, yet was not free of potential bias factors, such as the criteria used for selection of the animals. Obviously, it was not possible to standardize and uniform every step of the slaughtering phases, considering the gross differences between the religious and conventional slaughtering techniques. Regardless of this, we sampled the animals in three steps common to both the procedures and we did not alter/influence the execution of the various procedures. Accordingly, we only portrayed a picture of the actual differences existing between two animal groups representative of the two different procedures.

Recent market studies [37] reported that the perception of animal welfare has increased among European consumers, especially for the methods of breeding and transportation, introducing the possibility to use the claim “from certified herds”. On the other hand, there is no clear legislation for labeling of carcass judged as *Taref* (not suitable for consumption exclusively for religious reason), by Jewish religious slaughtering. In fact, this product is marketed without any kind of indication regarding the type of slaughtering, thus failing to protect the non-Jewish consumers [38].

Based on all the above considerations, stunning in ritual slaughtering was introduced in the UK in 2015. This virtuous model where religion and science meet up and find an agreement should be contemplated in the European legislation and exported to other European countries.

## Figures and Tables

**Table 1:** Mean values (M), Standard Deviation (SD) and Significance Level (*p*) between groups.

**(a) Plasma Cortisol**				
		**Cattle Farm (Step 1)**	**Lairage Facilities (Step 2)**	**Exsanguination (Step 3)**
Religious Jewish Rite	M	2.96	31.65	68.70
	SD	1.21	25.48	30.61
Traditional slaughter	M	4.85	36.36	45.08
	SD	3.23	12.21	14.15
Between groups	*p*	0.424	0.000	0.000
**(b) Plasma dopamine**				
Religious Jewish Rite	M	129.37	149.50	868.43
	SD	45.18	46.45	508.52
Traditional slaughter	M	132.47	172.10	513.87
	SD	64.56	61.21	286.32
Between groups	*p*	0.20	0.07	>0.02
**(c) Plasma norepinephrine**				
Religious Jewish Rite	M	273.87	478.47	3776.20
	SD	261.22	324.47	1918.44
Traditional slaughter	M	317.53	586.10	3425.57
	SD	104.93	284.66	1777.39
Between groups	*p*	0.78	0.09	>0.01
**(d) Plasma epinephrine**				
Religious Jewish Rite	M	198.47	275.67	4352.20
	SD	186.68	126.81	3730.15
Traditional slaughter	M	201.53	426.80	3279.97
	SD	81.59	341.01	1954.53
Between groups	*p*	0.93	0.31	0.17

**Table 2:** Mean values (M), Standard Deviation (SD) and Standard Error (SE) of the increase rates among the three steps. M from step 1 to step 2 expresses the difference between the parameter recorded in steps 2 and 1, divided by the level found in step 1. M from step 2 to step 3 expresses the difference between the parameter recorded in steps 3 and 2, divided by the level found in step 2. M from step 1 to step 3 expresses the difference between the parameter recorded in steps 3 and 1, divided by the level found in step 1.

**(a) Plasma Cortisol**				
		**From Step 1 to Step 2**	**From Step 2 to Step 3**	**From Step 1 to Step 3**
Religious Jewish Rite	M	11.96	1.88	25.51
	SD	12.95	2.33	15.31
	SE	2.37	0.43	2.79
Traditional slaughter	M	9.77	0.27	12.53
	SD	7.31	0.26	9.01
	SE	1.34	0.05	1.65
Total sample	M	10.87	1.08	19.02
	SD	10.49	1.83	14.07
**(b) Plasma dopamine**				
Religious Jewish Rite	M	0.30	5.25	6.72
	SD	0.63	4.01	5.67
	SE	0.11	0.73	1.03
Traditional slaughter	M	0.52	2.96	3.81
	SD	0.74	5.37	3.35
	SE	0.14	0.98	0.61
Total sample	M	0.41	4.11	5.27
	SD	0.69	4.84	4.84
**(c) Plasma norepinephrine**				
Religious Jewish Rite	M	1.21	8.57	17.31
	SD	1.28	4.76	9.86
	SE	0.23	0.87	1.80
Traditional slaughter	M	1.11	6.33	10.94
	SD	1.39	5.25	7.17
	SE	0.25	0.96	1.31
Total sample	M	1.16	7.45	14.12
	SD	1.32	5.09	9.13
**(d) Plasma epinephrine**				
Religious Jewish Rite	M	1.56	21.49	69.69
	SD	2.16	30.71	205.04
	SE	0.39	5.61	37.43
Traditional slaughter	M	1.62	14.93	17.16
	SD	2.60	17.51	11.40
	SE	0.47	3.20	2.08
Total sample	M	1.59	18.21	43.43
	SD	2.37	25.01	146.39
